# Thermal homogenization of boreal communities in response to climate warming

**DOI:** 10.1073/pnas.2415260122

**Published:** 2025-04-21

**Authors:** Jussi Mäkinen, Emilie E. Ellis, Laura H. Antão, Andréa Davrinche, Anna-Liisa Laine, Marjo Saastamoinen, Irene Conenna, Maria Hällfors, Andrea Santangeli, Elina Kaarlejärvi, Janne Heliölä, Ida-Maria Huikkonen, Mikko Kuussaari, Reima Leinonen, Aleksi Lehikoinen, Juha Pöyry, Anna Suuronen, Maija Salemaa, Tiina Tonteri, Kristiina M. Vuorio, Birger Skjelbred, Marko Järvinen, Stina Drakare, Laurence Carvalho, Erik Welk, Gunnar Seidler, Pieter Vangansbeke, František Máliš, Radim Hédl, Alistair G. Auffret, Jan Plue, Pieter De Frenne, Jesse M. Kalwij, Jarno Vanhatalo, Tomas Roslin

**Affiliations:** ^a^Research Center for Ecological Change, Organismal and Evolutionary Research Programme, Faculty of Biological and Environmental Sciences, University of Helsinki, Helsinki FI-00014, Finland; ^b^Nature Solutions Unit, Finnish Environment Institute (Syke), Helsinki FI-00790, Finland; ^c^Department of Biology, University of Turku, Turku FI-20014, Finland; ^d^Animal Demography and Ecology Unit, The Mediterranean Institute for Advanced Studies, Spanish National Research Council, University of the Balearic Islands, Esporles ES-07190, Spain; ^e^Kainuu Centre for Economic Development, Transport and the Environment, Kajaani FI-87101, Finland; ^f^Finnish Museum of Natural History, University of Helsinki, Helsinki FI-00014, Finland; ^g^Natural Resources Institute Finland, Helsinki FI-00790, Finland; ^h^Norwegian Institute for Water Research, Oslo NO-0579, Norway; ^i^Department of Aquatic Sciences and Assessment, Swedish University of Agricultural Sciences, Uppsala SE-75007, Sweden; ^j^Department of Geobotany and Botanical Garden, Martin Luther University, Halle-Wittenberg D-06099, Germany; ^k^Department of Environment, Forest & Nature Lab, Ghent University, Gontrode B-9090, Belgium; ^l^Department of Vegetation Ecology, Faculty of Forestry, Technical University in Zvolen, Zvolen SK-96053, Slovakia; ^m^Institute of Botany, Czech Academy of Sciences, Brno CZ-60200, Czech Republic; ^n^Department of Botany, Palacký University in Olomouc, Olomouc CZ-777900, Czech Republic; ^o^Department of Ecology, Swedish University of Agricultural Sciences, Uppsala SE-75007, Sweden; ^p^Department of Urban and Rural Development, Swedish University of Agricultural Sciences, Uppsala SE-75007, Sweden; ^q^Institute of Geography and Geoecology, Karlsruhe Institute of Technology, Karlsruhe D-76131, Germany; ^r^Department of Zoology, Centre for Ecological Genomics & Wildlife Conservation, University of Johannesburg, Auckland Park ZA-2006, South Africa; ^s^Department of Mathematics and Statistics, Faculty of Science, University of Helsinki, Helsinki FI-00014, Finland; ^t^Faculty of Biological and Environmental Sciences, Research Center for Ecological Change, Ecosystems and Environment Research Programme, University of Helsinki, Helsinki FI-00014, Finland

**Keywords:** community temperature index, niche position, niche breadth, climate change, climatic debt

## Abstract

Globally, rising temperatures are increasingly favoring warm-affiliated species. Although changes in community composition are typically measured by the mean temperature affinity of species (the community temperature index, CTI), they may be driven by different processes and accompanied by shifts in the diversity of temperature affinities and breadth of species thermal niches. To resolve the pathways to community warming in Finnish flora and fauna, we examined multidecadal changes in the dominance and diversity of temperature affinities among understory forest plant, freshwater phytoplankton, butterfly, moth, and bird communities. CTI increased for all animal communities, with no change observed for plants or phytoplankton. In addition, the diversity of temperature affinities declined for all groups except butterflies, and this loss was more pronounced for the fastest-warming communities. These changes were driven in animals mainly by a decrease in cold-affiliated species and an increase in warm-affiliated species. In plants and phytoplankton the decline of thermal diversity was driven by declines of both cold- and warm-affiliated species. Plant and moth communities were increasingly dominated by thermal specialist species, and birds by thermal generalists. In general, climate warming outpaced changes in both the mean and diversity of temperature affinities of communities. Our results highlight the complex dynamics underpinning the thermal reorganization of communities across a large spatiotemporal gradient, revealing that extinctions of cold-affiliated species and colonization by warm-affiliated species lag behind changes in ambient temperature, while communities become less thermally diverse. Such changes can have important implications for community structure and ecosystem functioning under accelerating rates of climate change.

## Significance

As climate warms, many species are shifting their distributions to track their climatic niches; this usually leads to a general increase in the mean temperature affinities of species within communities. Using monitoring data on species from terrestrial and freshwater realms in Finland, we show that most communities have become dominated by warm-affiliated species over time, with a parallel decrease in thermal diversity. These changes are driven in animals mainly by a decrease in cold-affiliated species and an increase in warm-affiliated species, while in plants and phytoplankton they were driven by consistent declines in both cold- and warm-affiliated species. Temperature-driven population dynamics can potentially have far-reaching impacts on these boreal communities and their ability to cope with further climate change.

Climate warming is driving many species ranges toward the poles (1, 2) and higher elevations (3) as species track climatically suitable areas. Climate-driven “community warming” (thermophilization) can simultaneously be driven by two population-level processes: increase in relatively warm-affiliated species, that is, species whose distributions are centered on areas warmer than the ranges of the other species considered (4–8), and decline in relatively cold-affiliated species (4). As population dynamics of both cold- and warm-affiliated species leave an imprint on community-level thermal composition, understanding the pathways underpinning the thermal reorganization of communities is fundamental for predicting the resilience of future ecosystems and for designing conservation strategies under climate change. For example, if the “warming” of communities is primarily driven by the decline of cold-affiliated species (9, 10), then this will identify the latter as being particularly at risk.

Any imbalance between the disappearance of cold-affiliated and colonization by warm-affiliated species will contribute to the diversity of temperature affinities and control the gap between the mean temperature affinity of a community and air temperature (11–13). Such dynamics may be coupled with changes in both functional and phylogenetic diversity, though concrete examples are rare (14). In general, we do not know how simultaneous changes in the populations of cold- and warm-affiliated species are currently impacting the thermal diversity of communities and the breadth of temperature conditions that species can inhabit. Although most previous studies focus on changes in communities’ average temperature affinities [that is, the community temperature index (CTI)] (9, 15), less is known about how the diversity of temperature affinities change (but see refs. 11 and 16). Temperature change may affect the relative abundances of species with wide vs. narrow thermal tolerances, i.e., “thermal generalists” and “thermal specialists,” respectively (17). The trajectories of these other dimensions of the temperature affinities represent important imprints of environmental change. Moreover, few studies have examined changes in thermal composition in both the terrestrial and freshwater realms (9), while boreal ecosystems are usually absent from these assessments, despite these regions warming faster than lower latitudes (18).

Here, we quantify community-wide changes in both the mean and diversity of temperature affinities over time, as well as shifts in the breadth of species temperature affinities, using abundance records from long-term monitoring data in Finland for 348 forest understory plant, 722 phytoplankton, 90 butterfly, 713 moth, and 145 bird species (see *SI Appendix*, Fig. S1 for the locations of the monitoring sites). We used species’ European ranges in relation to long-term temperature means from ref. 19 to calculate the mean and SD of species’ temperature affinities. We calculated the abundance-weighted CTI, that is, the mean temperature affinity of a community (15) based on the species temperature affinities (that is, the species temperature index; STI). In parallel, we measured the diversity of temperature affinities (CTIdiv) using the abundance-weighted SD of STIs within communities (15). To measure changes in the breadth of temperature affinities, we quantified the mean and diversity of the community temperature breadth index (CTBI and CTBIdiv, respectively). A species temperature breadth index (STBI) is the SD of the temperature values over the European range of the species. CTBI is defined as the abundance-weighted mean of the STBI in a given community, and CTBIdiv as the abundance-weighted SD of the STBI. Hence, CTBI corresponds to the average temperature breadth in a community, and CTBIdiv to the average deviation of a STBI from the community mean (see *STI and CTI* for mathematical definitions of temperature indices).

**Fig. 1.**
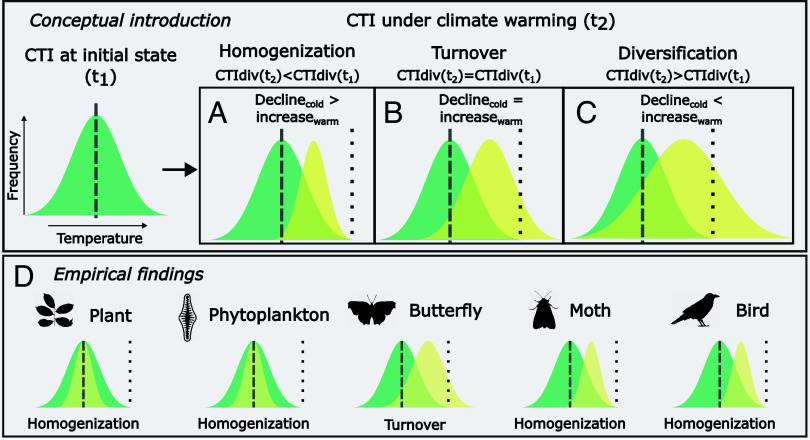
Contrasting pathways through changes in CTI and in the diversity of temperature affinities to community warming. (*A*–*C*) A conceptual representation of how the population dynamics of cold- and warm-affiliated species can lead to changes in CTI and CTIdiv (based on ref. 11). Distributions of species temperature affinities in an initial state (green) and under climate warming (yellow) are shown for different pathways; the dashed gray line denotes temperature at initial state, and dotted black lines denote temperature after climate warming. Across panels *A*–*C*, the mean values of the yellow distributions are the same, and only their width changes: with homogenization, a decline in cold-affiliated species increases CTI and decreases CTIdiv (*A*). With turnover, CTI increases, while CTIdiv stays constant (*B*). With diversification, an increase of warm-affiliated species increases CTI and widens CTIdiv (*C*)—note that the proportion of the distribution falling on the right-hand side of mean annual air temperature increases through an increase in CTIdiv. The *Bottom* panel *D* summarizes our empirical findings of increases and declines in CTI and CTIdiv across taxa.

To quantify the relative importance of cold- and warm-affiliated species in driving community warming, we further assessed changes in abundance trends for cold- and warm-affiliated species separately. To split species into a “cold” vs. a “warm” category, we used the median STI of each taxonomic group (see *SI Appendix*, Fig. S2 for the distributions and median of STI values of each taxonomic group). This allows us to pinpoint the drivers of homogenization, turnover, and/or diversification of temperature affinities in community change (Fig. 1, following refs. 10 and 11). In a situation where cold-affiliated species decline and warm-affiliated species show no change, we should observe an increase in CTI but a decrease in CTIdiv, indicating homogenization of temperature affinities (Fig. 1*A*). If cold-affiliated species tend to disappear at the same rate as warm-affiliated species colonize, we expect an increase in CTI but no changes in CTIdiv (Fig. 1*B*)—as these dynamics create turnover of temperature affinities. Finally, if warm-affiliated species increase faster than cold-affiliated species disappear, then we should observe increases in both CTI and CTIdiv (Fig. 1*C*) and hence thermal diversification.

Overall, we expected that CTI increases and CTIdiv decreases over time given rapid climate warming resulting from a faster decline in cold-affiliated species compared to the increase in warm-affiliated species (12, 13). These dynamics would also lead to an increasing gap between the mean annual air temperature and the CTI (2, 12). Among taxonomic groups, we expected a faster increase in CTI of highly mobile species (such as birds) than among sessile species (such as plants), as the former are able to track suitable conditions more efficiently than the latter, as well as for ectothermic species (such as butterflies and moths) compared to endothermic species (such as birds) (2). Such differences should arise because ectotherms should be less able to cope with increasing ambient temperatures through physiological mechanisms than endotherms (20). In addition, CTI should increase at a faster pace toward the poles, as climate warming is faster at higher latitudes, thus likely increasing the potential for colonizations by warm-affiliated species and extirpation of cold-affiliated ones (21). Finally, we expected CTBI to increase with time, reflecting increases in thermal generalists and a higher survival rate of more thermally flexible species (17, 22).

To test our hypotheses and to resolve changes in CTI, CTIdiv, CTBI, and CTBIdiv over time and space, we built spatially explicit statistical models for each metric and taxonomic group, thereby estimating the average rate of change in the response variable per year. Our approach integrates over the extinction and colonization events and population dynamics of individual species and provides a comprehensive assessment of community dynamics under climate warming.

## Results

### Results for CTI and CTIdiv.

**Fig. 2.**
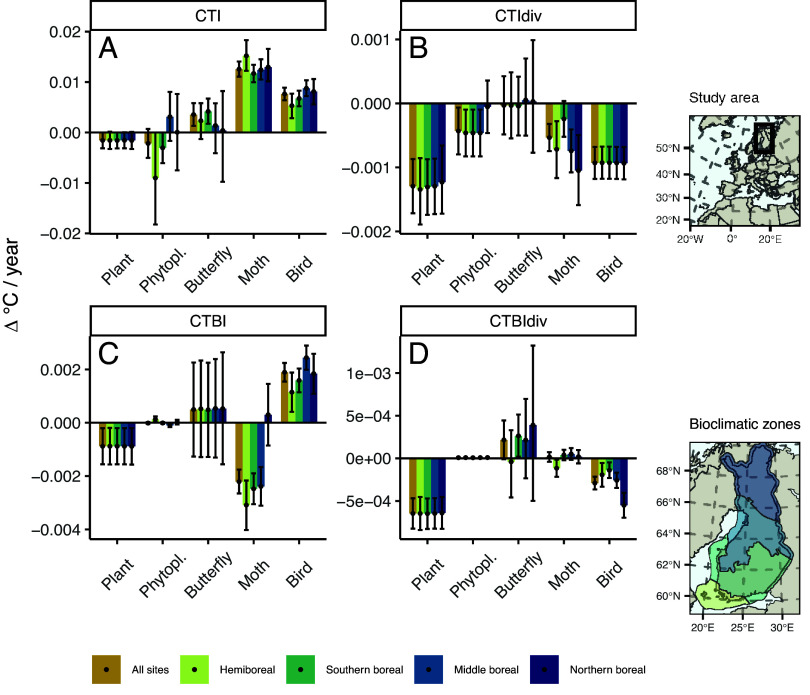
All animal groups have warmed while CTI of plants and phytoplankton have not changed. The composition of temperature affinities of all taxonomic groups except butterflies has become more homogeneous. Panels show the average annual change of temperature affinity distributions in Finnish taxa for CTI (*A*), CTIdiv (*B*), CTBI (*C*), and CTBIdiv (*D*). The bars and dots denote the average estimated change in Δ C^°^/year, while the error bars denote the 95% credibility interval for all sites and for each bioclimatic zone separately. The plots on the right show the study area and the geographical distribution of the bioclimatic zones.

For all animal groups, CTI increased with time, with moths showing the highest increase (butterflies: *β* = 0.004; moths: *β* = 0.013; birds: *β* = 0.008; all *P* < 0.05; where *β* equals the average annual rate of change in CTI estimated over monitoring sites). As expected, a temporal trend of CTI was not supported for plants or phytoplankton (both *P* > 0.05; Fig. 2). In terms of diversity of temperature affinities, CTIdiv decreased for all taxa (plants: β=−0.001; phytoplankton: β=−0.0004; moths: β=−0.0005; birds: β=−0.0009; all *P* < 0.05; Fig. 2), except butterflies (*P* > 0.05; Fig. 2). Despite the long latitudinal gradient over Finland and distinct bioclimatic zoning, the CTI and CTIdiv trends did not change directionally along the bioclimatic gradient for any taxa (Fig. 2).

**Fig. 3.**
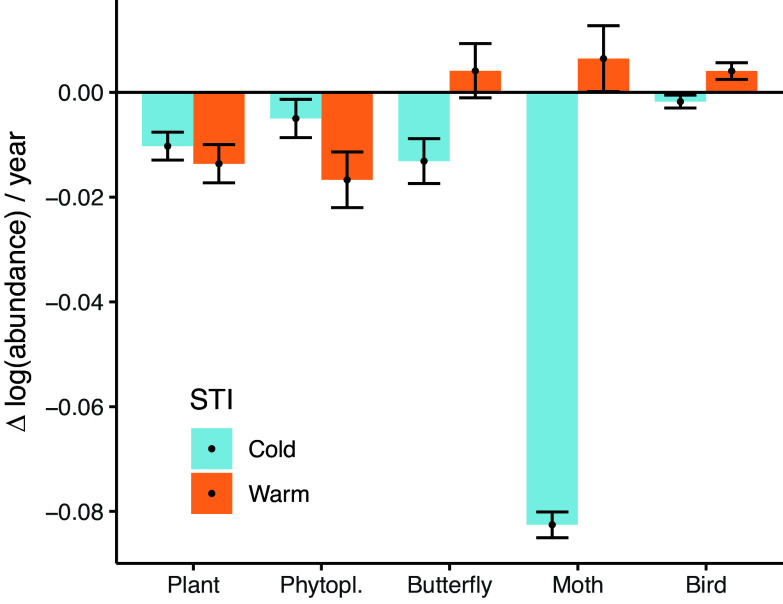
For plants and phytoplankton, cold- and warm-affiliated species have declined. For animal groups, cold-affiliated species have declined, while for moths and birds, warm-affiliated species have increased. Plot shows the changes in abundance of cold- and warm-affiliated species. Species are divided to cold- and warm-affiliated groups with respect to the taxonomic group–specific median STI value. Bars and dots denote the average estimated change in Δ log(abundance)/year, with error bars denoting the 95% credibility interval. The difference between cold- and warm-affiliated species is statistically significant for all taxonomic groups (*P* < 0.05).

Plants and phytoplankton decreased in the abundance of cold- and warm-affiliated species (Fig. 3). The decline rate was −0.01 for cold-affiliated and −0.014 for warm-affiliated plants measured as individuals on a logarithmic scale per year (both *P* < 0.05). For phytoplankton, the respective rates were −0.005 for cold-affiliated and −0.017 for warm-affiliated species (both *P* < 0.05). For all animal groups, we found a decrease in cold-affiliated species. Among moths and birds, we found an increase in warm-affiliated species. For butterflies, the rate was −0.013 (*P* < 0.05) for cold-affiliated and uncertain (*P* > 0.05) for warm-affiliated species. For moths, the rate was −0.083 (*P* < 0.05) for cold-affiliated and 0.006 (*P* < 0.05) for warm-affiliated species. For birds, the rate was −0.002 (*P* < 0.05) for cold-affiliated and 0.004 (*P* < 0.05) for warm-affiliated species. For all taxonomic groups, the difference in the rate of change for cold- and warm-affiliated species was statistically significant. For animal groups specifically, the difference between the absolute rates of change of cold- and warm-affiliated species was also statistically significant. This indicates that for butterflies and moths, the cold-affiliated species declined faster than the warm-affiliated increased, whereas for birds, the warm-affiliated species increased faster than the cold-affiliated declined.

**Fig. 4.**
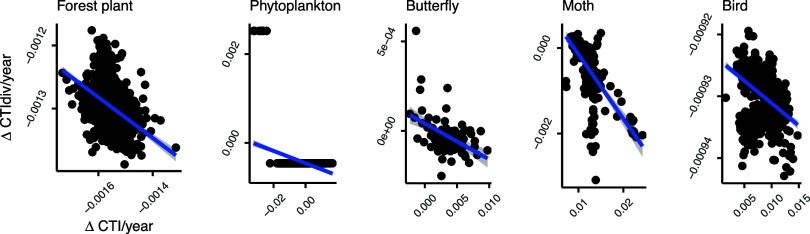
For every taxonomic group, the monitoring sites which warm the fastest also lose diversity of temperature affinities the fastest. Scatter plots show the relationship between temporal changes in CTI and CTIdiv. Each dot denotes an individual monitoring site, and the blue line shows a linear regression fitted for each taxonomic group (the shaded area denotes the 95% credibility interval).

Monitoring sites where communities were warming the fastest also showed the fastest decline in CTIdiv in all taxonomic groups [Fig. 4; Pearson’s correlation coefficients between CTI and CTIdiv: −0.39 for plants, −0.35 for phytoplankton, −0.47 for butterflies, −0.67 for moths, and −0.44 for birds (all *P* < 0.05)].

**Fig. 5.**
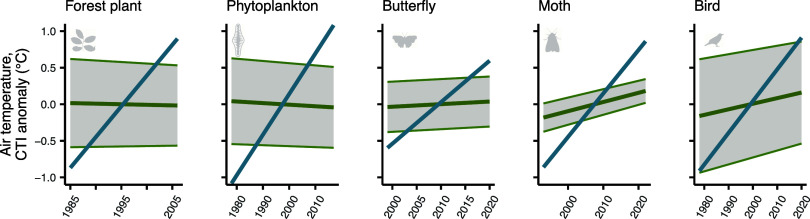
In every taxonomic group, mean annual air temperature increases faster than CTI, and exceeds the 95% of the temperature affinities of the communities. Temporal trajectory of CTI (green line), with the shaded area around CTI denoting the 95% quantile of the temperature affinities of the communities (calculated by multiplying CTIdiv by two) and the mean annual air temperature (blue line) during the monitoring period for each taxonomic group. All trajectories are drawn on the basis of the model estimates for the average change. For CTI and mean annual air temperature, the values on the y axis have been scaled to denote anomalies (that is, deviations from the mean over the monitoring period), since CTI values for all taxonomic groups were consistently 3 ^°^C higher than mean annual air temperature. Note that air temperature increases at somewhat different rates for different taxonomic groups, due to the spatiotemporal sampling design, as each taxonomic group is monitored in different sets of sites and for different time periods.

The rate of increase in mean annual air temperature exceeded the rate of increase in CTI in all taxonomic groups, being on par with or exceeding the average diversity of temperature affinities in a community (Fig. 5).

### Moth Population Dynamics.

The change in abundance of cold-affiliated moths was sensitive to abundance variation in one particularly dominant species, Epirrita autumnata (Geometridae), which exhibits cyclical population dynamics in northern Finland (23). A peak in the abundance of *E. autumnata* during the early part of our study period resulted in a strong and negative trend over years. To resolve the impact of this pattern, we repeated all analyses with *E. autumnata* excluded. This resulted in a minor shift in the rate estimate of cold-affiliated species, to −0.038 (*SI Appendix*, Fig. S3). As estimates of temporal rates of change in CTI and other community metrics were largely unaffected by the exclusion of *E. autumnata*, our overall conclusions are robust to variation in this specific species (*SI Appendix*, Fig. S3).

### Results for CTBI and CTBIdiv.

The mean breadth of temperature affinities, CTBI, increased for birds (*β* = 0.002, *P* < 0.05) but declined for plants and moths (plants: β=−0.0009; moths: β=−0.002; both *P* < 0.05), and remained stable for phytoplankton and butterflies (Fig. 2). The diversity of temperature affinity breadths (CTBIdiv) declined for plants and birds (β=−0.0006; β=−0.0003, respectively; both *P* < 0.05), while there was no statistically significant change over time for the other taxonomic groups.

## Discussion

### Taxonomic Groups Warm and Cool Differently.

As expected, CTI increased for animal groups with good dispersal abilities, while there was no change for plants and phytoplankton. Moreover, the rate of increase in CTI for moths outpaced that of birds, followed by butterflies, partially supporting the expectation that ectotherms respond faster than endotherms to climate warming. For moths and birds, the increase of CTI was driven by decreasing cold-affiliated and increasing warm-affiliated species. For butterflies, the rate of change of warm-affiliated species was uncertain. Our results support earlier studies showing that community warming results from the simultaneous decrease in cold-affiliated species and the increase in warm-affiliated species (6).

Here, we expanded the view on CTI change from the diversity perspective. We expected that differences in the paces at which cold- and warm-affiliated species decline or increase impact the trend of diversity of temperature affinities (CTIdiv). For moths and butterflies, we found a faster decline in cold-affiliated species compared to an increase in warm-affiliated species. These results showed that while the ranges of cold-affiliated species are contracting, some warm-affiliated species may not be able to sustain their expanding populations (1, 24). In fact, range expansion may be limited by habitat availability (12) and/or phenological mismatches, which for moths is caused by high annual variability in the onset of the growing season in northern Finland (21). However, only for moths this led to a decline in CTIdiv, as for butterflies, the estimate for the rate of change of CTIdiv was highly uncertain. For birds, we found that warm-affiliated species increased faster than cold-affiliated species decreased, which should lead to increase of CTIdiv. However, we detected a decline in CTIdiv. The discrepancy between expected and realized trajectory of CTIdiv for birds and butterflies, is likely caused by more complex population dynamics among our broad classification of warm- and cold-affiliated species, as shown by Santangeli et al. (25). For instance, these dynamics could be related to strong declines in only a few warm-affiliated species or strong increases in a few cold-affiliated species.

The plant and phytoplankton communities did not show a detectable change in CTI but a decline in CTIdiv. These CTI level dynamics were underpinned by the decrease in cold- and warm-affiliated species. Although we found a faster decrease in warm-affiliated species for plants and phytoplankton, these trends were balanced enough not to lead to changes in CTI, but strong enough to narrow the distribution of temperature affinities — a pattern previously reported for plants in refs. 26–28. For plants, we expect these results to be driven by forest succession. Our analyses were based on systematic sampling of plants in the forest understory, with the monitoring sites covering different successional stages. Ruderal forest plants consisting of both cold- and warm-affiliated species are on average characteristic of open and/or early-successional forest landscapes. With the closure of the canopy, such species will decline, to be replaced by plants with a preference for cool, shady microhabitats, whereas in older forests species abundance dynamics are somewhat static. This explains the relatively faster decrease of warm-affiliated plants. In addition, plant responses to mesoclimate warming can be slowed by microclimatic variability, which sustains cool microhabitats (29, 30).

Similarly to plants, for phytoplankton, a stagnant CTI can be attributed to buffering effects of habitat, as local factors in water bodies can override the effects of air temperature on phytoplankton communities (31, 32), as well as of the medium, since water requires relatively more energy per unit to warm than air (1 cal/^°^C/g). Thus, changes in the temperature of water bodies are less pronounced than changes in the surrounding atmosphere (33). Furthermore, other important factors affect phytoplankton communities, such as nutrients, alkalinity, and water color (34). These factors may also be affected by agriculture and the management of surrounding forests, which modify the physical and chemical conditions in lakes (35). Thus, both climatic buffering and local physico-chemical impacts beyond temperature can explain the simultaneous declines of cold- and warm-affiliated species and hence the counterintuitive association between changes in CTI and mean annual air temperature.

### Relative Composition of Thermal Specialists and Generalists.

Global change may result in an increase in generalist species at the expense of diminishing specialist species (17, 22). In our study, only birds showed this pattern of increased CTBI. This pattern was coupled with a decline in the diversity of temperature breadths (CTBIdiv). By contrast, plant and moth communities became more dominated by species with narrow thermal breadth over time. Moths and birds with narrower temperature affinities have been found to expand their ranges further north in Finland compared to species with broader temperature affinities (36)—a pattern that only partially coincides with our findings. Plants also lost diversity in terms of the breadth of temperature affinities. For plants, simultaneous declines in the mean and diversity of thermal breadths were likely due to forest succession and the increasing proportion of cold-affiliated specialist species in communities.

### Climatic Debt Depends on Both the Mean and Diversity of Temperature Affinities.

Given how quickly and consistently mean annual air temperatures have increased compared to CTI and CTIdiv, our findings show that the composition of species’ temperature affinities is not keeping up with the current rate of climate warming—and that this rift is emerging across a wide range of taxa in a boreal ecosystem. A generally assumed mechanism behind such “a climatic debt” is that cold-affiliated species decrease or go locally extinct faster than warm-affiliated species colonize, due, e.g., to dispersal barriers and habitat quality (6, 12, 24). Here, the climatic debt of butterflies and moths accumulates through such a process, whereas the climatic debt of plants and phytoplankton accumulates through simultaneous declines of cold- and warm-affiliated species.

Climatic debt through inadequate community warming can be a signal of current temperature-related processes and may have cascading ecosystem-level effects. In this paper, we have shown that communities that are warming the fastest also lose diversity of temperature affinities the fastest. If a decline in the diversity of temperature affinities is coupled with a decline in other facets of functional diversity (though not supported in ref. 14), then community warming can be linked to eroding ecosystem functions. Given a lack of knowledge regarding how species’ temperature affinities translate to their functional traits, we are unable to predict how this will affect community functioning. Future studies should address how the observed changes in the diversity of temperature affinities are matched by changes in the community-level distribution of other functional traits. For instance, differences in the pace of community warming among taxonomic groups can cause rifts in the distribution of interaction partners, e.g., between insectivorous birds and their prey (9, 37), thus affecting species interactions. Such temperature-driven effects can further cascade into diminishing population abundances and taxonomic and functional diversity. Indeed, we found differences in the climatic debt exhibited by different taxonomic groups—but demonstrating that these findings translate to changes in species interactions would require being able to map the changes observed onto species groups of known interactions.

We did not detect consistent differences along bioclimatic gradients in our analysis, despite ample variation between individual monitoring sites in terms of community warming. This variability between sites within regions can probably be traced to different land use trajectories and to the fact that the sites vary in their current protection status (25). Indeed, it is worth noting that different ecosystems and geographical regions vary in their rates and extent of community warming, with important implications for regional conservation priorities (13, 15). This variability between geographical regions can be due to opposing trends in ambient temperature change (15), different levels of species sensitivity to climate warming (15), or buffering effects of habitat characteristics (such as canopy cover) to warming temperatures (26). Our study shows that climate warming is outpacing the diversity of temperature affinities created by historical and current biogeographical processes. Thus, establishing a well-designed network of protected areas—or using measures such as habitat restoration to increase habitat variability (16, 38)—could contribute to communities keeping up with climate warming and sustain healthy ecosystems.

### CTI for Capturing Temperature-Driven Community Change.

An important consideration when assessing our current findings is how the metrics of species’ temperature affinities were derived: they are measures of climatic conditions within the species distributions in Europe (following an established practice of refs. 9 and 24), not thermal limits established through physiological experiments. In addition, this realized range is also affected by factors other than temperature, such as precipitation, land use, and/or soil type, while different populations may have varying degrees of local adaptation to these factors. Hence, STI is not a direct measure of species temperature optima, but a proxy derived from the geographic subset where the other requirements of the species are met, while the STBI may be affected by variability among and between populations. Thus, caution should be taken when interpreting these estimates of temperature affinities, as they do not necessarily match precisely the temperature optimums and tolerance measured by other means. The same caveats should be considered when assessing climatic debt (39). Nevertheless, these metrics offer a useful proxy for temperature preferences when analyzing large-scale patterns for many species and taxa (40, 41). In addition, we did not consider precipitation, which has a strong impact on species performance and persistence for many taxonomic groups. In particular, precipitation can interact with temperature in affecting the thermal regulation of animals and soil moisture accessible to plants (42, 43). However, for boreal vegetation and ecosystems, the temperature exceeds precipitation as the limiting factor, and therefore we can expect that our analysis reveals the main axis of community warming (44). The absence of precipitation should not affect our current conclusions about changes in CTI and other temperature metrics. Instead, it would at most increase the random variation in the final models. Therefore, we consider our broad definition of climate debt (CTI—ambient temperature) (6, 11) a useful measure of how communities track climate. Together, our findings of temperature-driven changes in community composition point to further changes to come as climate change accelerates.

### Data Archival.

The species range maps used for computing STI are partly copyright material and therefore cannot be included in the original format along with the publication. However, the same maps can be accessed through the references cited in the paper: for all butterflies and some moths from refs. 45 and 46, for the rest of the moths from refs. 47–67, for all plants from refs. 68–73, and for birds from ref. 60. For phytoplankton, we used distributional data from the WISER database based on Europe-wide monitoring data (74). Unfortunately, the data have not been opened as proposed in that publication, and thus we refer the reader to database manager Birger Skjelbred (birger.skjelbred@niva.no) for data queries.

The monitoring datasets are openly available for moths (https://laji.fi/en/observation/list?collectionId=HR.4511), birds (https://laji.fi/observation/list?target=MX.37580&collectionId=HR.61), and phytoplankton (https://www.syke.fi/en/environmental-data/open-web-services/environmental-data-apis#phytoplankton). For butterflies, the data are not directly available as the data users need to commit to the user policies of the eBMS network. However, the data are accessible as part of a wider European butterfly transect dataset through eBMS after formally completing a license form (https://butterfly-monitoring.net/ebms-data%20access). For plants, the data are not publicly available but can be inquired from Tiina Tonteri (tiina.tonteri@luke.fi) and Maija Salemaa (ext.maija.salemaa@luke.fi).

Exclude this sentence, as it is only for peer-review. All data of species temperature indices (except for birds) and community temperature indices allowing readers to repeat the analysis will be shared along with the publication in https://doi.org/10.5061/dryad.4j0zpc8np.

## Materials and Methods

To comprehensively address the responses of thermal community composition to climate warming, we used long-term community monitoring data and species range maps. Our spatially extensive (340,000 km^2^) and multidecadal (22 to 42 y, depending on the taxonomic group) data on species abundances document the nationwide dynamics of understory forest plant, phytoplankton, butterfly, moth, and bird communities in Finland. We used Europe-wide range maps for deriving species-specific temperature affinities, which we then used to calculate community-level metrics for CTI, CTIdiv, CTBI, and CTBIdiv. To assess the pace at which communities have changed, we fit spatially explicit statistical models for each metric and estimated the rate of change in the response variable per year. We investigated regional patterns by mapping the observed rates to bioclimatic zones within Finland [hemiboreal, southern boreal, middle boreal, and northern boreal; (75)]. To assess the local rate at which climatic debt may be accumulating, we fit a similar model for the mean annual air temperature and compared the rates of change between CTI and CTIdiv with the mean annual air temperature (6, 11).

### Species and Climate Data.

To estimate temporal trends, we used species data from Finnish community monitoring programs. For all groups except butterflies, the sampling covers the whole of Finland (60^°^N to 70^°^N, 19.4^°^E to 31^°^E) and all its bioclimatic zones. For butterflies, the monitoring program centers on agricultural landscapes in Southern and Central Finland. For each taxonomic group, the survey methodology follows a standardized protocol.

For birds, we used records collected in 1978–2020, including 145 species sampled across 1,149 transects (76, 77). The transects are 3 to 6 km long and are visited once a year during the survey. Surveys are typically conducted in June, with some variation in exact dates due to the latitudinally varying phenology of the breeding season. Although not every transect is surveyed each year, survey intensity (walking speed, survey methodology, and width of the area surveyed around a transect) is constant over surveys. The data were curated and processed by the Finnish Museum of Natural History. We derived STIs for all bird species.

For butterflies, records were collected in 1999–2020 and included 91 species sampled across 101 transects (78, 79). Transects are typically focused on agricultural landscapes. The surveys are carried out by volunteers and the monitoring program is directed by the Finnish Environment Institute (Syke). Not every transect is surveyed each year. Surveys are conducted at least seven times per site during May to August. Given latitudinal gradients in the length of the season, the survey period varies from 10 wk in northern Finland to 16 wk in southern Finland. The survey intensity of each single transect visit is kept constant between sites and years, and the species identification skill of the volunteers is high. We derived STIs for 90 butterfly species.

For moths, records were collected 1993–2023 and included 713 species of macromoths sampled in 246 traps. The moth recording is conducted under the National Moth Monitoring scheme (Nocturna) and coordinated by Syke (80, 81). Light traps are run from April to October/November in the south of Finland and May to September/October in the north, thus covering the entire activity period of moths. The traps are emptied on average weekly by volunteers and the identification of moth catches is also done by volunteers with quality control by the coordination team. The data have been curated by monitoring coordinators.

For plants, 1,700 sample sites were monitored in 1985–1986 and 1995, with a resurvey of 443 sites in 2006. All sites are located on mineral soils. At each site, the percentage cover of vascular forest plants (small tree and shrub seedlings and saplings, dwarf shrubs, herbs, and graminoids) was recorded in four 2 m^2^ quadrats systematically located three, six, and eight meters away from the center of the plot (82, 83). The sites were located in clusters of four sites, 16 km apart from each other in southern Finland, and clusters of three sites, 24 km apart in the east-west direction and 32 km apart in the north–south direction in northern Finland. In total, 487 vascular forest plant species were recorded. The data are curated and maintained by the Natural Resources Institute Finland (Luke). We derived STIs for 348 plants as some species were missing a range map.

Phytoplankton records were collected 1978–2017 in 853 lakes at 1,057 study sites. In total, 1,222 species were detected. The surveys were carried out from early July to late August, reflecting the peak productivity season of lake phytoplankton communities. All phytoplankton samples were preserved with acid Lugol’s solution and analyzed using the standard Utermöhl technique (84). Phytoplankton communities have been monitored in inland and coastal waters by national environmental authorities for assessments of the state of the water bodies. The dataset is curated and maintained by Syke (https://ckan.ymparisto.fi/dataset/kasviplanktontietojarjestelma-kplank). We derived STIs for 722 species.

For all groups except for plants, the starting point and duration of surveys vary between sites—which we explicitly accounted for in our models discard this. For all groups, the effort per annual survey is fixed, except for birds for which the survey transects can vary in length between survey sites. However, since the transect length is not associated with an environmental factor or cluster in space or time, the variability in the transect length creates uncertainty, but not a systematic bias in our model estimates.

We derived mean annual air temperature data for the monitoring sites based on a daily record of mean temperature provided by the Finnish Meteorological Institute (85) at a resolution of 10 × 10 km. For annotating European range maps with long-term climatic means, we used annual mean temperature over 1961–1990 as available from ref. 19.

### STI and CTI.

STIs were derived from the European ranges of species following the approach of ref. 45. For birds, we adopted the range maps of BirdLife’s range map catalog (60). For all butterflies and some moths, range maps were derived from the ALARM project (45, 46). For most of the moths, range maps were digitized from refs. 47–67 and for all plants from refs. 68–73. For phytoplankton, we used distributional data from the WISER database based on Europe-wide monitoring data (74). This dataset is collated from monitoring programs from 26 European countries for management purposes and is described in detail in ref. 74.

For all species except phytoplankton, we had access to range maps and could thus derive STIs by overlaying range maps with climatic means over 1961–1990 (19). Species ranges from Atlas range maps were digitized and coarsened to the Common European Chorological Grid (CGRS) at a 50 × 50 km resolution. The climate data of Fronzek et al. (19) come in the same grid system provided by Schweiger et al. (45). We computed STI as the mean temperature value over the species range and STBI as the SD of temperature values over the species range. STI was used to derive CTI and CTIdiv and STBI was used to derive CTBI and CTBIdiv. As emphasized in the main text (CTI for Capturing Temperature-Driven Community Change), STI does not directly correspond to species’ temperature optima. This is due to the fact that other factors, such as precipitation and soil type, will also impact a species’ distribution. As such imprints of additional environmental variables are not taken into account in the estimate of STI, this metric will, strictly speaking, represent the temperature conditions within areas where other factors are also suitable for the species.

Because the range data for phytoplankton were based on survey data from unevenly distributed survey sites, we predicted species ranges throughout Europe by fitting a species distribution model for each species individually. We annotated plankton presence–absence observations at monitoring sites with climatic variables in 50 × 50 km resolution from ref. 19, fitted a generalized linear model and predicted species ranges across Europe using the same climate variables in 50 × 50 km resolution. Although phytoplankton surveys started in 1922, we only included records from 1980 onward, to ensure that survey methodologies have not changed during the survey period. We assume that the prevalence of species at a survey site is reflected in the rate at which they are detected. We set the response variable as the number of times a species is detected in a survey site and set the number of trials as the number of surveys conducted at a site. Assuming that climatic variables do not vary across the survey period, we could consider survey sites as response units and hence our sample size corresponded to the number of survey sites. We modeled the prevalence of species occurrence as binomially distributed and modeled the logit-transformed response as a function of first- and second-order effects of mean annual temperature, annual temperature range, annual precipitation sum, and annual precipitation range. After fitting a model, we predicted a species range across the cells in CGRS given their climatic conditions. We calculated the mean and SD for STI by taking the presence probability weighted mean and SD of the mean annual temperature over all cells in CGRS. Hence, we did not need to set a threshold to transform a predicted presence probability to a presence–absence status.

For all communities, we then calculated CTI as the abundance-weighted mean of STI $\mathrm{CTI}=\frac{\sum_{k=1}^{K}w_{k}STI_{k}}{\sum_{k=1}^{K}w_{k}}$, where *w*_*k*_ denotes the relative abundance of a species *k* in a community that has a species richness equal to *K*. We derived thermal diversity, CTIdiv, as the abundance-weighted SD of STI in a community $\mathrm{CTIdiv}=\sqrt{\frac{\sum_{k=1}^{K}w_{k}{\left(STI_{k}-CTI\right)}^{2}}{\left(K-1\right)\sum_{k=1}^{K}w_{k}}}$, the community temperature breadth index, CTBI, as the abundance-weighted mean of STBI $\mathrm{CTBI}=\frac{\sum_{k=1}^{K}w_{k}STBI_{k}}{\sum_{k=1}^{K}w_{k}}$ and CTBIdiv as the abundance-weighted SD of STBI $\mathrm{CTBIdiv}=\sqrt{\frac{\sum_{k=1}^{K}w_{k}{\left(STBI_{k}-CTBI\right)}^{2}}{\left(K-1\right)\sum_{k=1}^{K}w_{k}}}$.

### Inference Model.

To estimate the rates of change in thermal diversity and composition and assess climatic debt, we modeled the average change in mean annual air temperature, CTI, CTIdiv, CTBI, and CTBIdiv per year. To account for variation in the starting years and in the duration of the surveys across monitoring sites, we defined the temporal responses as spatially varying random effects. This allows responses to vary smoothly over neighboring sites and smooths out site-level differences (which partially originate from different sampling periods). The linear predictor for each response variable was defined as

$$y_{i}\left(x_{i}\right)=\alpha +\beta_{s_{i},0}+\beta_{s_{i}}*\;\text{year}+\varepsilon_{i},$$

where *y*_*i*_ is the *i*:th sample of the response variable, *α* is a model intercept, $\beta_{s_{i},0}$ is a spatially varying intercept, $\beta_{s_{i},\;\text{year}}$ is a spatially varying linear weight for the effect of year, and *ε*_*i*_ is a random error term.

We kept the response variables within their original ranges and set the explanatory variable for year to start from zero, thereby creating intuitive estimates for the *β* parameter—i.e., estimates of average change of the response variable per year in degrees Celsius. We also included a spatially varying random intercept in the models to account for other spatially structured variability.

#### Spatial random effects.

We defined spatially varying intercepts and responses to time as a Besag, York, and Mollié (BYM)-type random effect. The BYM-type effect accounts for the spatial structure by averaging the effect at a single monitoring site over the effects at neighboring sites (86). We used the scaled version of the BYM-type effect from ref. 87. Here, the response consists of two parts reflecting the unstructured and structured spatial effects of the monitoring site, respectively. Hence, it is defined as

$$\beta_{s_{i}}∼N\left(\frac{1}{\sqrt{\tau_{s}}}\left(\sqrt{1-\phi }v_{s_{i}}+\sqrt{\phi }u_{s_{i}}\right),\tau_{s}^{-1}\left(\left(1-\phi \right)I+\phi Q^{-}\right)\right),$$

where **v** is a spatially unstructured effect and **u** is a spatially structured effect, *τ*_*s*_ is a marginal variance parameter, *ϕ* is a mixing parameter, and $Q^{-}$ is the generalized inverse of the adjacency matrix. The mixing parameter varies between 0 and 1, reducing the effect to a pure overdispersion effect when *ϕ* = 0 and to a purely spatially structured effect when *ϕ* = 1. We assigned penalized complexity priors to *τ*_*s*_ and *ϕ* so that $P(\tau_{s})>1=0.01$ and P(ϕ)>0.5=0.5 (87). We defined the adjacency matrix with the k-nearest neighbor method by fixing the number of neighbors per site to six. We used the chooseCN-function of the adegenet R package (v. 2.1.8) for computing the adjacency matrix (88). To derive average temporal rate of change of the response variables over all monitoring sites or over bioclimatic zones we averaged $\beta_{s_{i}}$ according to the set considered. To derive comprehensive uncertainty estimates for the averaged effects, we took into account spatial covariance between sites.

All models were fitted with the INLA package (v. 22.05.07) in R (v. 4.2.1). INLA is specifically tailored for fitting hierarchical Bayesian models to large datasets. It allows estimating complex spatial and temporal dependencies in the data and alleviates the computational burden by approximating the marginal probability of the data with the Laplace approximation (89). Here, we took advantage of these properties in fitting multiple spatial random effects and in propagating uncertainty to areal averages of the random effects.

### Assessing Abundance Trends of Cold- and Warm–Affiliated Species.

To evaluate the temporal trends of different thermal groups, we used the STI values to classify species within each taxonomic group as being either cold- or warm–affiliated, using the median (50% quantile) as the threshold: cold (<50%) and warm (≥50%). Next, we summed the abundances over all species in a taxonomic group for both categories per site and year. Last, we fitted a spatially explicit statistical model of the abundance of cold- and warm-affiliated species. The models were similar to those used for CTI and the other community-level metrics. In addition, for plants and phytoplankton, observations reflect biomass, thus including continuous positive values with a high prevalence of zeros and a long tail toward large values. To account for these data characteristics, we fitted a log-normal model. For butterflies, moths, and birds, observations reflect the number of individuals (positive integers), and hence we applied a Poisson observation model with a logarithmic link function.

In addition, we simplified the spatial process for these models, by using a Besag-type spatial random effect, which has only the spatially structured part. While a BYM-type random effect (as used for the community-level models) provided a better fit for the data, it was overparameterized for the data at hand and resulted in unrealistic parameter estimates. For abundance models, applying a Besag-type random effect led to better convergence rates and more intuitive parameter estimates than a BYM-type random effect.

## Supplementary Material

Appendix 01 (PDF)

## Data Availability

All data of species temperature indices (except for birds) and community temperature indices along with accompanying code allowing readers to repeat the analysis will be shared along with the publication in https://doi.org/10.5061/dryad.4j0zpc8np (90). The species range maps used for computing species temperature indices are partly copyright material and therefore cannot be included in the original format along with the publication. However, the same maps can be accessed through the references cited in the paper. For phytoplankton, we used distributional data from the WISER database based on Europe-wide monitoring data. Unfortunately, the data have not been opened as proposed in that publication, and thus we refer the reader to database manager Birger Skjelbred (birger.skjelbred@niva.no) for data queries. The monitoring datasets are openly available for moths (https://laji.fi/en/observation/list?collectionId=HR.4511) (91), birds (https://laji.fi/observation/list?target=MX.37580&collectionId=HR.61) (92), and phytoplankton (https://www.syke.fi/en/environmental-data/open-web-services/environmental-data-apis#phytoplankton) (93). For butterflies, the data are not directly available as the data users need to commit to the user policies of the eBMS network. However, the data are accessible as part of a wider European butterfly transect dataset through eBMS after formally completing a license form (https://butterfly-monitoring.net/ebms-dataaccess) (94). For plants, the data are not publicly available but can be inquired from Tiina Tonteri (tiina.tonteri@luke.fi) and Maija Salemaa (ext.maija.salemaa@luke.fi).
