# Supplementary material for: Thermal homogenization of boreal communities in response to climate warming
Source: Proc Natl Acad Sci U S A. 2025 Apr 21;122(17):e2415260122. doi: 10.1073/pnas.2415260122 (PMC12054843; doi:10.1073/pnas.2415260122)
Supplement: Supplementary file 1 — Appendix 01 (PDF) [file pnas.2415260122.sapp.pdf]

Supporting Information for

Supporting information for *Thermal homogenization of boreal communities in response to climate warming*

Mäkinen et al.

Corresponding Author Jussi Mäkinen.
E-mail: jussi.makinen@helsinki.fi

This PDF file includes:

Figs. S1 to S3

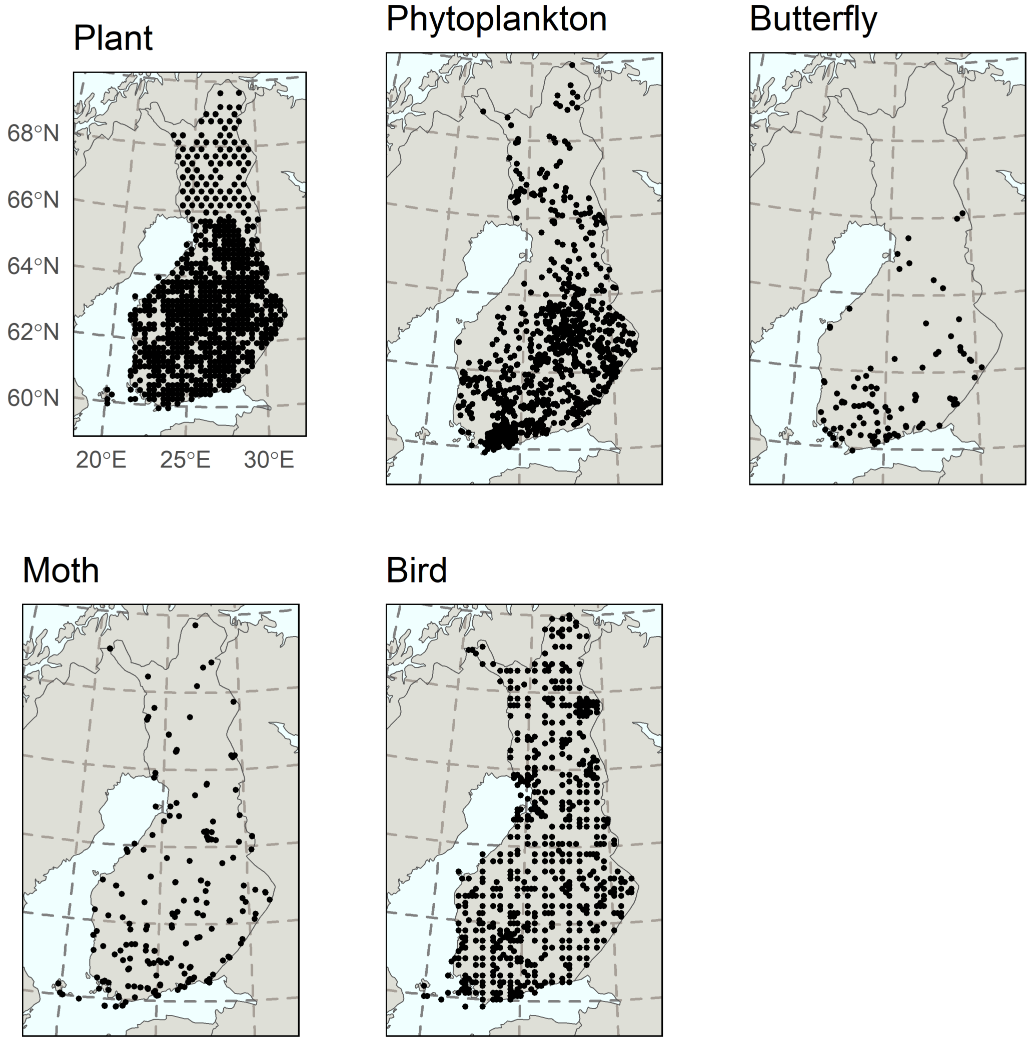

Fig. S1. Species monitoring sites. Each dot represents a unique monitoring site.

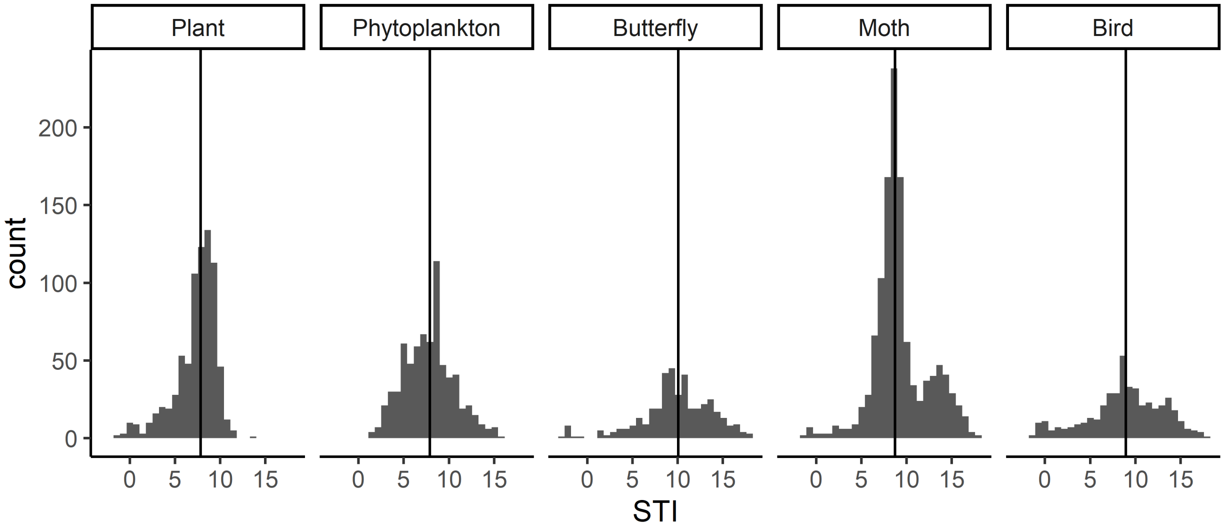

Fig. S2. Histograms show the distributions of STI values of each taxonomic group. Vertical lines denote the median STI values which are used to split species to cold and warm categories.

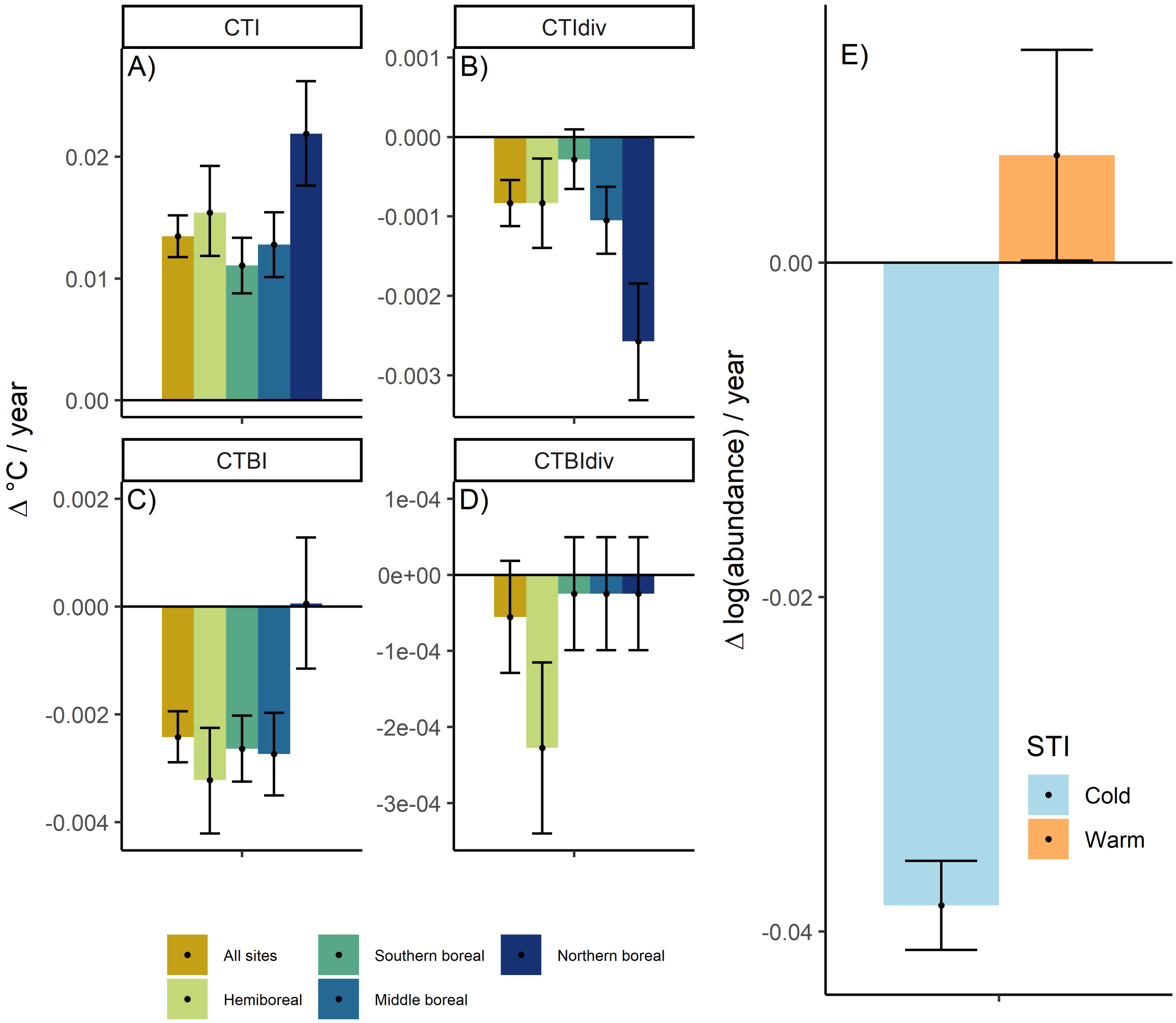

Fig. S3. Model estimates for the average annual change of temperature affinity distributions and species abundance in moths when excluding *Epirrita autumnata* (Geometridae) from the observation data. **Panels A-D** show show the average annual change of temperature affinity distributions in moths for mean temperature affinity (CTI; A), diversity of temperature affinities (CTIdiv; B), community temperature breadth index (CTBI; C) and diversity of temperature breadth index (CTBIdiv; D). The bars and dots denote the average estimated change in Δ C° / year, with the error bars denoting the 95 % credibility interval for all sites and for each bioclimatic zone separately. **Panel E** shows the changes in the abundance of cold- and warm-affiliated species for moths. Species are split to STI group/category with respect to median STI value of recorded moths. Bars and dots denote the average estimated change in Δ log(abundance)/year, with error bars denoting the 95 % credibility interval.
